# Construction of the 1,2-Dialkenylcyclohexane Framework via Ireland-Claisen Rearrangement and Intramolecular Barbier Reaction: Application to the Synthesis of (±)-Geijerone and a Diastereoisomeric Mixture with Its 5-Epimer

**DOI:** 10.3390/molecules19011238

**Published:** 2014-01-20

**Authors:** Dawei Liang, Nana Gao, Wei Liu, Jinhua Dong

**Affiliations:** Key Laboratory of Structure-Based Drug Design and Discovery, Ministry of Education, Shenyang Pharmaceutical University, Shenyang 110016, Liaoning, China; E-Mails: ldawei@yahoo.com (D.L.); gaonana89@126.com (N.G.); lwnever@126.com (W.L.)

**Keywords:** 1,2-dialkenylcyclohexane, (±)-geijerone, Ireland-Claisen rearrangement, intramolecular barbier reaction, synthesis

## Abstract

The elemene-type terpenoids, which possess various biological activities, contain a *syn*- or *anti*-1,2-dialkenylcyclohexane framework. An efficient synthetic route to the *syn*- and *anti*-1,2-dialkenylcyclohexane core and its application in the synthesis of (±)-geijerone and its diastereomer is reported. Construction of the *syn*- and *anti*-1,2-dialkenyl moiety was achieved via Ireland-Claisen rearrangement of the (*E*)-allylic ester, and the cyclohexanone moiety was derived from the iodoaldehyde via intramolecular Barbier reaction. The synthetic strategy allows rapid access to various epimers and analogues of elemene-type products.

## 1. Introduction

**Figure 1:**
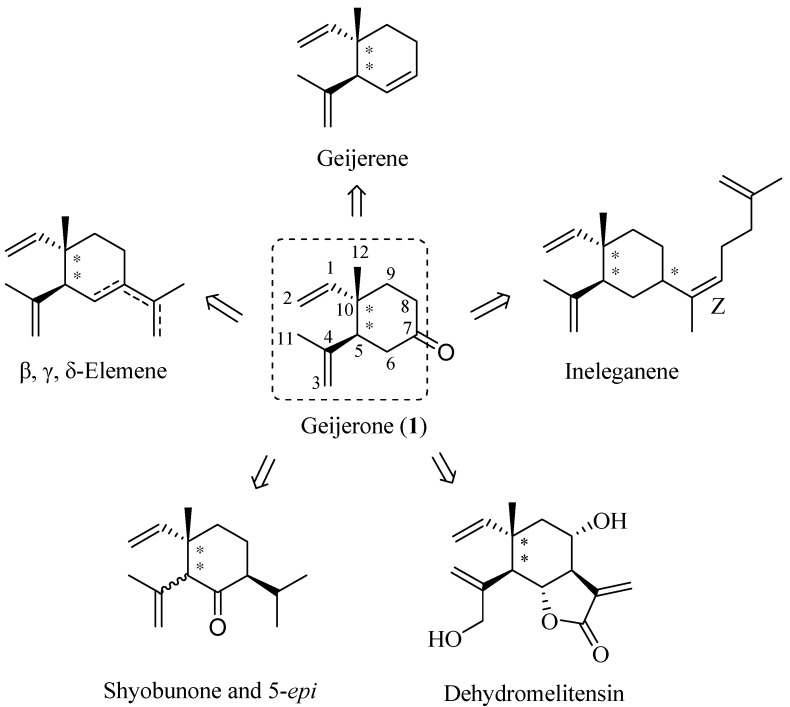
Structures of elemene-type terpenoids and (±)-geijerone (**1**).

Natural products continue to attract intense attention due to their various bioactivities and they have played a vital role in the field of drug discovery in recent decades. Most of the drugs in the clinical market today are inspired by or derived from natural sources [1]. β-Elemene, γ-elemene, δ-elemene, geijerene, ineleganene, shyobunone and dehydromelitensin are natural terpenoids with a *syn*- or *anti*-1,2-dialkenylcyclohexane skeleton (Figure 1), and exist in various essential oils [2,3,4,5,6,7,8,9]. These compounds or their racemic mixtures have been shown to inhibit tumor cell growth *in vitro* and *in vivo* [10,11,12,13,14,15]. The mixture of β-elemene, γ-elemene and δ-elemene has been put into clinical trials in cancer patients in China [16,17].

A lot of efforts have been made towards the synthesis of these compounds due to their specific structures and important biological activities. For instance, Wu’s group reported the synthesis of elemene derivatives starting from carvone, employing a double Michael reaction as the key step [18,19]. In addition, other colleagues have reported their synthetic strategies for the synthesis of β-elemene, including Cope rearrangement, Ireland-Claisen rearrangement, doubly diastereo-differentiating folding and allylic strain-controlled intramolecular ester enolate alkylation [20,21,22,23,24].

Structurally, (±)-geijerone (**1**, Figure 1) contains a highly functionalized *anti*-1,2-dialkenyl-cyclohexane moiety and the 7-carbonyl group of (±)-geijerone (**1**) is beneficial for derivatization reactions. Therefore, (±)-geijerone (**1**) could be considered as a common precursor in the synthesis of elemene-type terpenoids. Kim utilized an intramolecular ester enolate alkylation to construct (±)-geijerone (**1**) and synthesized ã-elemene by starting from a rare lactol [25]. Another synthesis of (±)-geijerone (**1**) was reported by Yoshikoshi, using the Wieland-Miescher ketone as the starting material [26]. As a part of our synthetic studies on direct construction of the *syn*- and *anti*-1,2-dialkenylcyclohexane skeleton and bioactive elemene-type terpenoids we describe herein a novel and alternative synthesis of a mixture of two diastereomers of (±)-geijerone (**1**) by starting from the chainlike and commercially available geraniol (**2**).

## 2. Results and Discussion

**Scheme 1:**
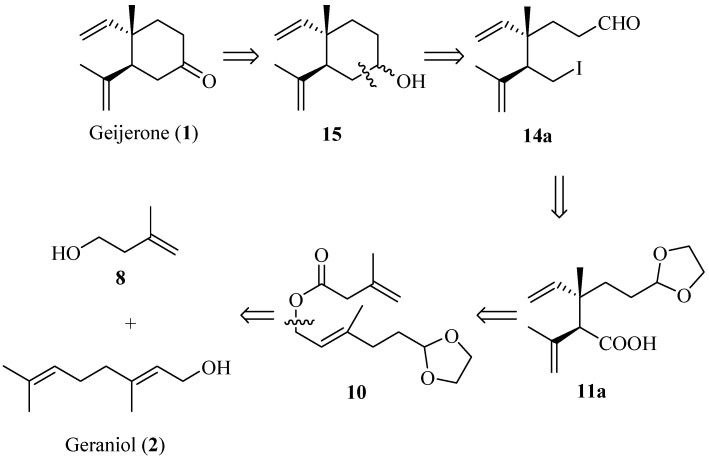
Retrosynthetic analysis of (±)-geijerone (**1**).

The retrosynthetic analysis is outlined in Scheme 1. (±)-Geijerone could be synthesized from **14a** via intramolecular Barbier reaction and subsequent oxidation. The conversion of **11a** to **14a** could be achieved by conventional methods. The *anti*-1,2-dialkenyl carboxylic acid **11a** could be constructed from (*E*)-allylic ester **10** using an Ireland-Claisen rearrangement as the key step. The ester 10 could be derived from geraniol (**2**) and 3-methyl-3-buten-1-ol (**8**).

**Scheme 2:**
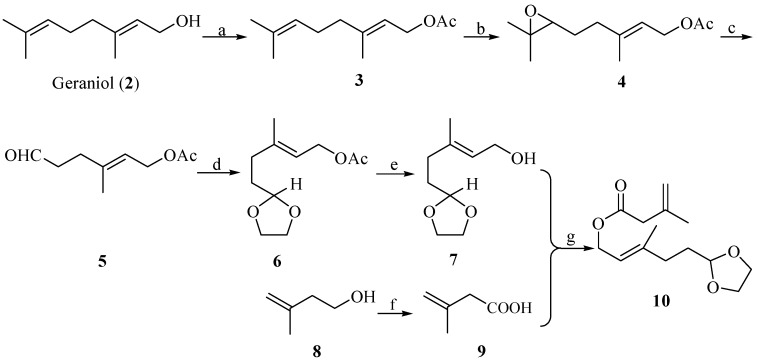
Construction of the key intermediate (*E*)-allylic ester **10**.

Our synthesis commenced with the construction of the key (*E*)-allylic ester intermediate **10** (Scheme 2). Protection of the hydroxyl group in geraniol (**2**) by acetyl chloride in pyridine gave **3** in 84% yield. Selective epoxidation of **3** at the double bond between C-6, C-7 with *m*-chloro- peroxybenzoic acid afforded **4 **(72%), and the ring cleavage reaction was undertaken with periodic acid to afford aldehyde **5** (80%) [27,28]. Next, protection of the aldehyde group of **5** gave acetal **6** in 90% yield, and removal of the acetyl group of **6** with anhydrous potassium carbonate afforded alcohol **7** in 82% yield. The oxidation of **8** to acid **9** (58%) was achieved with Jones’ reagent. Finally, the subsequent esterification reaction of **7** and **9** in the presence of 1-(3-dimethylaminopropyl)-3-ethyl-carbodiimide hydrochloride (EDCI) (1 equiv.) and 4-dimethylaminepyridine (DMAP) (0.05 equiv.) gave the desired ester **10** in 42% yield.

**Scheme 3:**
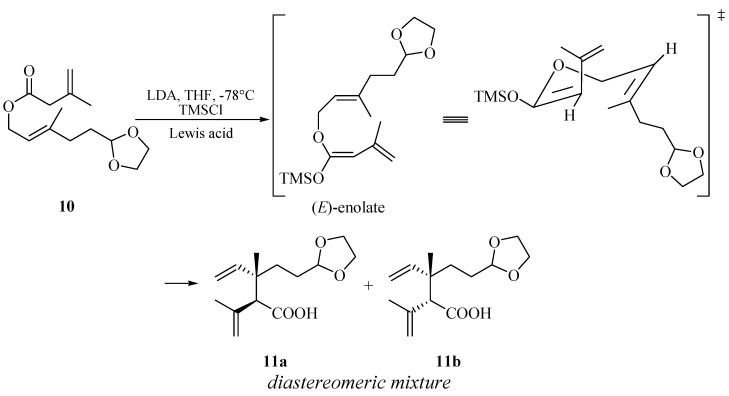
Proposed advantage transition state of acid **11** from ester **10** via Ireland-Claisen rearrangement.

With ester **10** in hand, our first challenge was to construct the 1,2-dialkenyl moiety. We chose to accomplish this goal by the Ireland-Claisen rearrangement strategy. The rearrangement of ester **10** to acid **11** was conducted with lithium diisopropylamide (LDA) (2 equiv.) and chlorotrimethylsilane (TMSCl, 2 equiv.) at −78 °C in anhydrous tetrahydrofuran, followed by a conventional operation. A proposed mechanism according to Ireland-Claisen rearrangement was outlined in Scheme 3 [29]. The formation of preferential configuration of the (*E*)-silyl enol ether could help to rationalize the possible six-membered, acyclically advantage chair-like transition state. Further the [3,3]-sigmatropic rearrangement of the (*E*)-silyl enol ether afforded the *syn*- and *anti*-1, 2-dialkenyl moiety in acid **11** as a mixture inseparable by silica gel chromatography. From the ^1^H-NMR results, the diastereomeric ratio of *anti* **11a**/*syn* **11b** could be readily deduced from the double-double signals for the vinyl proton at δ 5.84/6.05 (*J* = 10.8, 17.5 Hz, -CH=). These ^1^H-NMR results were in agreement with those reported in the literature [21]. Koch *et al.* have demonstrated that the use of Lewis acid results in a highly diastereoselective rearrangement of allylic esters [30]. Thus, by using this protocol, we closely investigated the application of several Lewis acid catalysts to optimize the Claisen–Ireland rearrangement, and the results were summarized in Table 1. It was found that the *anti* diastereomer **11a** was the major product (dr = 2:1, entry 1) when no Lewis acid catalyst was used, while the presence of various Lewis acids was unfavorable for improving the diastereoselectivity in this substrate. Other possible conditions to improve the diastereoselectivity were not screened. 

*Reagents and conditions*: (a) CH_3_COCl, pyridine, 0 °C–r.t. (84%); (b) *m*-CPBA, CH_2_Cl_2_, −5–0 °C (72%); (c) HIO_4_·2H_2_O, THF, Et_2_O, 0 °C (80%); (d) glycol, benzene, *p*-TsOH(cat.), reflux, 5 h (90%); (e) K_2_CO_3_ (cat.), CH_3_OH, r.t., 12 h (82%); (f) Jones reagent (58%); (g) EDCI+DMAP (cat.), CH_2_Cl_2_, r.t, 12 h (42%).

**Table 1:** Lewis acid-catalyzed Ireland-Claisen rearrangement of ester **10**.

Entry ^a^	Lewis acid	Yield(%) ^b^	dr ^c^
1	None	72%	2:1
2	TMSOTf	94%	1:1
3	BF_3_-Et_2_O	24%	1:1
4	ZnCl_2_	75%	1:1
5	SnCl_4_	91%	1:1

^a^ Reagents: Ester **10**: LDA: TMSCl: Lewis acid = 1.0 equiv.: 2.0 equiv.: 2.0 equiv.: 0.1 equiv.; ^b^ Isolated yields from **10**;^ c^ Diastereomeric ratio (*anti*/*syn*) was calculated by ^1^H-NMR analysis of the purified mixture **11**.

**Scheme 4:**
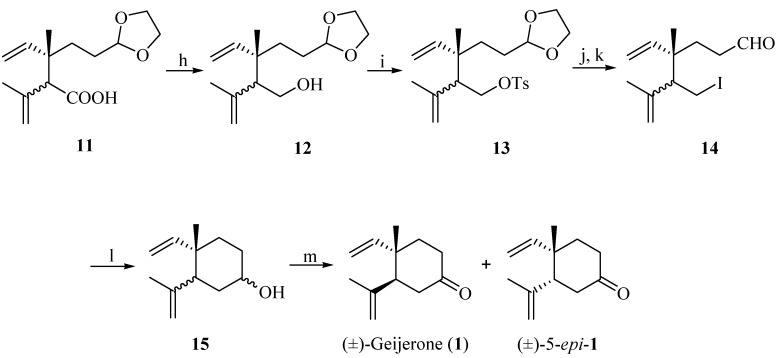
Synthesis of (±)-geijerone (**1**) and a diastereoisomeric mixture with its 5-epimer.

Subsequenr reduction of acid **11** with lithium aluminium hydride (LiAlH_4_) gave a diastereomeric mixture of alcohols **12** in 83% yield. The diastereomeric separation of the mixture of alcohols was attempted by esterification of **12** with chiral *O*-acetyl mandelic acid, but the result was not ideal. Treatment of **12** with tosyl chloride gave a mixture of sulfonic esters **13**, which after iodination and subsequent acetal deprotection provided a mixture of diastereoisomeric compounds **14**, which was unstable during long-term storage. The diastereoisomers of **11**–**14** were difficult to separate by silica gel column chromatography, and various attempts to achieve the separation of the diastereoisomers using an appropriate chromatographic column size were undertaken. These methods have not been successful so far and the diastereomeric ratio of **14** was only raised to 3:1 (by ^1^H-NMR analysis). Subsequently, the cyclization of **14** occurred in the presence of *n*-BuLi via intramolecular Barbier reaction [31,32,33,34,35,36] to give a diastereomeric mixture of alcohols **15** (38%). The direct addition of *n*-BuLi to **14** might be the cause for the low yield. Further optimization of this reaction is not described in this communication. Finally, compound **15** was oxidized by pyridinium chlorochromate (PCC), affording a mixture of **1** and its 5-epimer (60%) (Scheme 4).

*Reagents and conditions*: Reagents and conditions: (h) LiAlH_4_, THF, 0 °C–r.t. and reflux (83%); (i) *p*-TsCl, CH_2_Cl_2_, pyridine, r.t.; (j) NaI, acetone, reflux in the dark for 40 h; (k) *p*-TsOH (cat.), acetone (10%H_2_O), 1 h (23%, 3 steps); (l) *n*-BuLi, −78 °C (38%); (m) PCC, CH_2_Cl_2_, r.t., 2 h (60%).

## 3. Experimental

### General Information

Several commercially available solvents were dried by standard procedures before use: pyridine (NaOH), THF (Na), acetonitrile (CaCl_2_). Other commercial sources were used without further purification. ^1^H-NMR and ^13^C-NMR spectra were recorded with Bruker ARX-300 (300 MHz for ^1^H-NMR and 150 MHz for ^13^C-NMR) and Bruker ARX-600 spectrometers using TMS as internal standard (chemical shifts in δ values, *J* in Hz). Low-resolution MS and high-resolution MS data were obtained on Agilent-6120 Quadruple LC/MS and Agilent-6520 QTOF LC/MSD spectrometer, respectively, using ESI ionization. Column chromatography was performed on silica gel (200–300 mesh, Qingdao Haiyang Chemical Co., Ltd, Qingdao, China). Analytical TLC was performed on plates precoated with silica gel (GF254, 0.25 mm, Qingdao Haiyang Chemical Co., Ltd.) and iodine vapor was used to develop color on the plates.

*3-Methyl-3-butenoic Acid* (**9**). To a stirred solution of **8** (6.0 g, 69.7 mmol) in acetone (200 mL) the Jones reagent (36.5 mL, 97.5 mmol) was dropwise added at 0 °C for 2 h, and the resulting mixture was stirred at room temperature for another 6 h. The reaction mixture was quenched with H_2_O (50 mL), and most of acetone was evaporated under reduced pressure. The residue was extracted with Et_2_O (3 × 20 mL), The combined ethereal solution was washed with saturated aqueous NaHCO_3_ solution. The combined aqueous layer was acidified with diluted hydrochloric acid (2 M) to pH = 3, and then extracted again with Et_2_O (2 × 20 mL). All combined ethereal solution was washed successively by water, brine and dried (anhydrous MgSO_4_), concentrated in *vacuo*. The residue was distilled to give 3.3 g (58%) of acid **9**, colorless oil, b.p. = 86–88 °C (25 mmHg).^ 1^H-NMR (300 MHz, CDCl_3_): δ = 10.14 (s, 1H, -COOH); 4.96 (dd, *J* = 1.5, 10.8 Hz, =CH_2_); 3.09 (s, 2H, -CH_2_-); 1.84 (s, 3H, -CH_3_).

*(E)-3,7-Dimethylocta-2,6-dienyl Acetate* (**3**). To a stirred solution of geraniol (**2**, 30.0 g, 194.8 mmol) in pyridine (80 mL) was added dropwise CH_3_COCl (16.5 mL) at 0 °C over 2 h, and the resulting mixture was stirred at room temperature for 3 h. The reaction mixture was poured to dilute hydrochloric acid solution (5%, 500 mL) and stirred for 30 min. The aqueous layer was extracted by EtOAc (3 × 30 mL) and the combined organic phases were washed successively by saturated aqueous NaHCO_3_, brine and dried (anhydrous MgSO_4_), The solution was concentrated under reduced pressure to yield 36.5 g (84%) acetate **3**, colorless oil. ^1^H-NMR (300 MHz, CDCl_3_): δ = 5.37–5.32 (m, 1H, =CH-), 5.10-5.06 (m, 1H, =CH-), 4.59 (d, 2H, *J* = 7.2 Hz, -CH_2_O-), 2.12-2.01 (m, 7H, -CH_2_CH_2_-, CH_3_CO-), 1.70 (s, 3H, -CH_3_), 1.68 (s, 3H, -CH_3_), 1.60 (s, 3H, -CH_3_). ESI-MS (*m/z*): 219.2 (M+Na)^+^.

*(E)-5-(3,3-Dimethyloxiran-2-yl)-3-methylpent-2-enyl Acetate* (**4**). A solution of *m*-chloroperbenzoic acid (85%, 18.1 g) in CH_2_Cl_2_ (160 mL) was added to a solution of **3** (15.0 g, 76.5 mmol) in CH_2_Cl_2_ (235 mL) at −5 °C over 2 h. The resulting mixture was stirred at 0 °C for 2 h. The white precipitate was formed during the reaction (mainly *m*-chlorobenzoic acid). The reaction mixture was diluted with saturated aqueous NaHCO_3_ (250 mL) and the aqueous layer was extracted with CH_2_Cl_2_ (2 × 30 mL). The combined organic layer was washed with saturated aqueous NaHCO_3_, brine and dried (anhydrous MgSO_4_), and concentrated under reduced pressure. The residue was chromatographed on silica gel using 10% EtOAc/petroleum ether, affording 11.8 g (72%) of **4** as a colorless oil. ^1^H-NMR (300 MHz, CDCl_3_): δ = 5.42–5.37 (m, 1H, =CH-), 4.59 (d, 2H, *J* = 7.2 Hz,-CH_2_O-), 2.70 (t, 1H, *J* = 6.3 Hz, oxirane-H ), 2.26–2.13 (m, 2H, -CH_2_-), 2.05 (s, 3H, -CH_3_CO-), 1.73 (s, 3H, -CH_3_), 1.70–1.63 (m, 2H, -CH_2_-), 1.31 (s, 3H, -CH_3_), 1.26 (s, 3H, -CH_3_). ESI-MS (*m/z*): 235.2 (M+Na)^+^.

*(E)-3-Methyl-6-oxohex-2-enyl acetate* (**5**). To a stirred solution of **4** (5.0 g, 23.6 mmol) in Et_2_O (80 mL) was added dropwise HIO_4_∙2H_2_O (5.8 g, 25.4 mmol) in THF (50 mL) at 0 °C over 2 h. The resulting mixture was stirred at 0 °C for 3 h. Then the reaction mixture was diluted with water (100 mL) and the aqueous layer was extracted with Et_2_O (2 × 30 mL). All organic phases were combined and washed successively by saturated aqueous NaHCO_3_, brine and dried (anhydrous MgSO_4_), and concentrated under reduced pressure. The residue was chromatographed on silica gel using 10% EtOAc/petroleum ether giving 3.2 g (80%) of **5** as a colorless oil. ^1^H-NMR (300 MHz, CDCl_3_): δ = 9.78 (s, 1H, -CHO), 5.39–5.34 (m, 1H, =CH-), 4.59 (d, 2H, *J* = 6.9 Hz, -CH_2_O-), 2.61–2.56 (m, 2H, -CH_2_-), 2.40–2.36 (m, 2H, -CH_2_-), 2.05 (s, 3H, CH_3_CO-), 1.73 (s, 3H, -CH_3_). ESI-MS (*m/z*): 193.1 (M+Na)^+^.

*(E)-5-(1,3-Dioxolan-2-yl)-3-methylpent-2-enyl Acetate* (**6**). A mixture of **5** (5.0 g, 29.4 mmol), ethylene glycol (2.7 g, 43.5 mmol), and *p*-TsOH (0.1 g, 0.6 mmol) in benzene (80 mL) was heated at reflux for 5 h. After the reaction was quenched with saturated NaHCO_3_ solution (40 mL) and the solvent was evaporated, the residue was partitioned between EtOAc (3 × 200 mL) and saturated aqueous NaCl (2 × 100 mL). The organic layer was dried over anhydrous MgSO_4_ and evaporated to give a residue which was purified by silica-gel chromatography (hexanes/EtOAc = 100:1) to afford **6** (5.7 g, 90%) as a yellow oil. ^1^H-NMR (300 MHz, CDCl_3_): δ = 5.40–5.35 (m, 1H, =CH-), 4.86 (t, 1H, *J* = 4.8 Hz, -OCH (-O-)CH_2_-), 4.58 (d, 2H, *J* = 7.2 Hz, -CH_2_O-), 3.99–3.94 (m, 2H, -OCH_2_-), 3.87–3.82 (m, 2H, -OCH_2_-), 2.17 (t, 2H, *J* = 8.1 Hz, -CH_2_-), 2.05 (s, 3H, CH_3_CO-), 1.82–1.75 (m, 2H, -CH_2_-), 1.72 (s, 3H, -CH_3_). ESI-MS (*m/z*): 237.1 (M+Na)^+^.

*(E)-5-(1,3-Dioxolan-2-yl)-3-methylpent-2-en-1-ol* (**7**). A mixture of **6** (5.0 g, 23.4 mmol), and anhydrous K_2_CO_3_ (0.7 g, 5.0 mmol) in CH_3_OH (100 mL) was stirred at room temperature for 12 h (monitored by TLC, R*_f_* = 0.5, EtOAc/petroleum ether = 1:1). Most of the solvent was evaporated *in vacuo*, the residue was diluted with water (30 mL) and extracted with Et_2_O (3 × 20 mL). The ethereal solution was combined and washed brine, dried (anhydrous MgSO_4_) and concentrated *in vacuo* to give the crude product, which was chromatographied over silica gel (EtOAc/petroleum ether, 20:80 → 40:60) to afford 3.4 g (82%) of **7** as a yellow oil. ^1^H-NMR (300 MHz, CDCl_3_): δ = 5.46–5.41 (m, 1H, =CH-), 4.86 (t, 1H, *J* = 4.7 Hz, -OCH(-O-)CH_2_-), 4.13 (d, 2H, *J* = 6.9 Hz, -CH_2_O-), 3.99–3.95 (m, 2H, -OCH_2_-), 3.87–3.83 (m, 2H, -OCH_2_-), 2.15 (t, 2H, *J* = 8.0 Hz, -CH_2_-), 1.85–1.75 (m, 2H, -CH_2_-), 2.05 (s, 3H, CH_3_CO-), 1.69 (s, 3H, -CH_3_). ESI-MS (*m/z*): 195.0 (M+Na)^+^.

*(E)-5-(1,3-Dioxolan-2-yl)-3-methylpent-2-enyl 3-methylbut-3-enoate* (**10**). A mixture of **7** (1.7 g, 10.0 mmol), **9** (1.0 g, 10.0 mmol), DCC (2.3 g, 12.0 mmol) and DMAP (0.3 g, 2.5 mmol) in CH_2_Cl_2_ (30 mL) was stirred at room temperature for 12 h (monitored by TLC, R*_f_* = 0.8, EtOAc/petroleum ether = 1:5). The white precipitate was filtered, and the filtrate was washed successively by HCl (5%), saturated NaHCO_3_, brine, dried (anhydrous MgSO_4_) and concentrated *in vacuo* to give crude product, which was purified by silica-gel chromatography (EtOAc/petroleum ether, 5:95) to afford 1.1 g (42%) of **10** as light yellow oil. ^1^H-NMR (300 MHz, CDCl_3_): δ = 5.40–5.35 (m, 1H, =CH_2_), 4.91–4.62 (m, 3H, =CH_2_, =CH-, -OCH(-O-)CH_2_-), 4.62–4.60 (d, 2H, *J* = 6.9 Hz, -CH_2_O-), 3.99–3.95 (m, 2H, -OCH_2_-), 3.87–3.82 (m, 2H, -OCH_2_-), 3.03 (s, 2H, -CH_2_CO-), 2.17 (t, 2H, *J* = 8.1 Hz, -CH_2_-), 1.82 (s, 3H, -CH_3_), 1.80-1.75 (m, 2H, -CH_2_-), 1.72 (s, 3H, -CH_3_). ^13^C-NMR (150 MHz, CDCl_3_): δ = 16.4, 22.3, 31.9, 33.5, 43.3, 61.3, 64.8, 103.9, 114.5, 118.4, 138.5, 141.4, 171.2. ESI-MS (*m/z*): 277.1 (M+Na)^+^. HRMS (ESI): calcd for C_14_H_23_O_4_ (M+H)^+^, 255.1596, found 255.1603.

*(3S)-3-(2-(1,3-Dioxolan-2-yl)ethyl)-3-methyl-2-(prop-1-en-2-yl)pent-4-enoic Acid* (**11**). A solution of 2 equiv. of LDA in dry THF (3 mL, 2 M) was cooled to −78 °C. To this stirred solution was added 1.0 equiv. of the ester **10** (0.76 g, 3 mmol), dropwise over 10 min. Following the addition, the reaction mixture was stirred at −78 °C for 10 min and the 2 equiv. of Me_3_SiCl (0.76 mL, 6 mmol) in THF (6 mL) was added dropwisely to the reaction mixture for 10 min. The reaction mixture was stirred at −78 °C for 1.5 h and allowed to warm to 25 °C for 2 h. After the reaction was quenched with 1 N NaOH (20 mL) and the organic phase was washed with 1 N NaOH (15 mL), the aqueous phases were combined and acidified by HCl (5%) to pH = 1. the mixture was extracted with Et_2_O (3 × 15 mL), dried with anhydrous MgSO_4_, filtered and concentrated *in* *vacuo* to afford the crude acid, which was purified by silica gel chromatography (5% MeOH/CH_2_Cl_2_) to give 0.55 g (72%) of **11** as an inseparable mixture of diastereomers (dr = 2:1), as a light yellow oil. ^1^H-NMR (300 MHz, CDCl_3_): δ (mixture of two diastereomers) = 6.05 and 5.84 (dd, 1H, *J* = 10.8, 17.5 Hz, -CH=), 5.12–4.95 (m, 4H, =CH_2_, =CH_2_), 4.82 (t, 1H, *J* = 2.1 Hz, -OCH(-O-)CH_2_-), 3.97–3.89 (m, 2H, -OCH_2_-), 3.85–3.81 (m, 2H, -OCH_2_-), 3.06 (s, 1H, 4-H), 1.85 (s, 3H, -CH_3_), 1.69–1.55 (m, 4H, -CH_2_CH_2_-), 1.16 and 1.11 (s, 1H, -CH_3_). ^13^C-NMR (150 MHz, CDCl_3_): δ = 19.4, 23.8/24.5, 28.8, 32.5/33.4, 42.0, 61.2/61.8, 63.6/64.8, 104.8, 114.0/114.3, 117.1/117.5, 139.7, 143.2/143.5, 177.4. HRMS (ESI): calcd for C_14_H_23_O_4_ (M+H)^+^, 255.1596, found 255.1564.

*(3S)-3-(2-(1,3-Dioxolan-2-yl)ethyl)-3-methyl-2-(prop-1-en-2-yl)pent-4-en-1-ol* (**12**). To a stirred solution of LiAlH_4_ (1.5 g, 39.3 mmol) in dry THF (15 mL) was added dropwise the inseparable mixture of **11** (2.0 g, 7.9 mmol) in dry THF (20 mL) at 0 °C over 10 min. The reaction mixture was stirred at room temperature for 5 h and allowed to warm to reflux for 1 h. The mixture was quenched successively by H_2_O (1.5 mL), NaOH (15%, 1.5 mL) and 4.5 mL H_2_O on the ice-bath condition and stirred at room temperature for 30 min. The white precipitation was filtered and washed with Et_2_O (2 × 10 mL). All the organic phases were combined, dried (anhydrous MgSO_4_), filtered and concentrated *in* *vacuo* to give the crude product, which was purified by silica gel chromatography (EtOAc/petroleum ether, 5:95) to afford 1.58 g (83%) of **12** as an inseparable mixture of diastereomers (dr = 2:1), light yellow oil. R*_f_* = 0.35 (EtOAc/petroleum ether, 1:3). ^1^H-NMR (300 MHz, CDCl_3_): δ (mixture of two diastereomers) = 5.77 and 5.72 (dd, 1H, *J* = 10.8, 17.5 Hz, -CH=), 5.11–4.79 (m, 5H, =CH_2_, =CH_2_, -OCH(-O-)CH_2_-), 3.95–3.93 (m, 2H, -OCH_2_-), 3.85–3.83 (m, 2H, -OCH_2_-), 3.77–3.59 (m, 2H, -CH_2_OH), 2.25–2.22 (m, 1H, 4-H), 1.79 and 1.79 (s, 3H, -CH_3_), 1.57–1.48 (m, 4H, -CH_2_CH_2_-), 1.03 and 0.98 (s, 1H, -CH_3_). ^13^C-NMR (150 MHz, CDCl_3_): δ = 17.7, 25.3, 28.5, 35.4, 41.1, 60.8/61.9, 62.4, 64.9/65.4, 102.2, 110.3, 113.1, 144.7, 149.7. HRMS (ESI): calcd for C_14_H_25_O_3_ (M+H)^+^, 241.1804, found 241.1794.

*(3S)-5-(Iodomethyl)-4,6-dimethyl-4-vinylhept-6-enal* (**14**). To a stirred solution of the inseparable mixture of **12** (1.3 g, 5.4 mmol) in dry pyridine (20 mL) was added dropwise tosyl chloride (1.5 g, 7.9 mmol) in CH_2_Cl_2_ (15 mL) at ice-water temperature over 10 min. Then the reaction mixture was stirred at room temperature for additional 12 h. The reaction mixture was quenched with 10% HCl (30 mL) and extracted with CH_2_Cl_2_ (3 × 15 mL). All the organic phases were combined and washed successively by saturated NaHCO_3_, brine, and dried over anhydrous MgSO_4_, filtered and concentrated *in* *vacuo* to give the crude product **13**, which was used directly in the next step without further purification. R*_f_* = 0.60 (EtOAc/petroleum ether, 1:3). A solution of the inseparable mixture of **13**, NaI (3.9 g) in acetone (25 mL) was allowed to reflux for 40 h in dark condition. After the reaction mixture was cooled to room temperature, the precipitation was filtered and concentrated to give brown oil, which was added saturated Na_2_S_2_O_3_ (30 mL) and extracted with ether (3 × 15 mL). The combined organic phases were washed by brine and dried over anhydrous MgSO_4_, filtered and concentrated *in* *vacuo* to give the crude iodide product, R*_f_* = 0.80 (EtOAc/petroleum ether, 1:5). Then the solution of the crude iodide product, *p*-TsOH (0.1 g) in 10%H_2_O/acetone (10 mL) was allowed to reflux for 2 h. Most of acetone was evaporated *in* *vacuo*, the residue was diluted with saturated NaHCO_3_, and extracted with ether (3 × 10 mL). The combined ether was washed by brine, dried over anhydrous MgSO_4_, filtered and concentrated to give the crude product, which was purified by silica gel chromatography (EtOAc/petroleum ether, 2:98) to afford inseparable mixture of **14** (0.38 g, 23% in 3 steps). light yellow oil. R*_f_* = 0.50 (EtOAc/petroleum ether, 1:8). Trials were performed to establish the separability of the diastereoisomers using an appropriate chromatographic column size. These attempts have not been successful so far and the diastereomeric ratio of **14** was only raised to 3:1. ^1^H-NMR (300 MHz, CDCl_3_): δ (mixture of two diastereomers) = 5.71 and 5.64 (dd, 1H, *J* = 10.8, 17.4 Hz, -CH=), 5.22-4.79 (m, 4H, =CH_2_, =CH_2_), 3.53–3.49 (m, 1H, -CH_2_I), 3.15–3.06 (m, 1H, -CH_2_I), 2.45–2.30 (m, 3H, -CH_2_CHO and 5-H), 1.65–1.82 (m, 5H, -CH_2_-, -CH_3_), 1.04 and 0.97 (s, 1H, -CH_3_). HRMS (ESI): calcd for C_12_H_20_IO (M+H)^+^, 307.0559, found 307.0941.

*(±)-Geijerone (1) and its 5-epimer*. A solution of the inseparable mixture of **14** (0.5 g, 1.6 mmol) in dry THF (10 mL) was cooled to −78 °C. To this stirred solution was added dropwise 3.0 equiv. of *n*-BuLi (1.6 M/L in *n*-hexane, 3.1 mL, 4.8 mmol) over 10 min. The reaction mixture was stirred at −78 °C for additional 1 h. Then the reaction mixture was quenched with saturated NH_4_Cl (30 mL), and the organic phase was separated. The aqueous phase was extracted with Et_2_O (3 × 10 mL). All organic phases were combined and washed by brine and dried over anhydrous MgSO_4_, filtered and concentrated *in* *vacuo* to afford inseparable diastereomeric mixture of crude **15**, light yellow oil. HRMS (ESI): calcd for C_12_H_21_O (M+H)^+^, 181.1592, found 181.1584.

A solution of the inseparable mixture of **15** (70 mg, 0.6 mmol) in 10 mL of CH_2_Cl_2_ was added dropwise PCC (0.5 g, 2.3 mmol) in CH_2_Cl_2_ (10 mL) at room temperature over 10 min, and the reaction mixture was stirred at room temperature for additional 2 h. Then the reaction mixture was diluted with 10 mL of diethyl ether, filtered and concentrated *in* *vacuo* to give the crude product, which was purified by silica gel chromatography (EtOAc/petroleum ether, 2:98) to afford an inseparable diastereomeric mixture of **1** and its 5-epimer (40 mg, 60%), colorless oil. R*_f_* = 0.75 (EtOAc/petroleum ether, 1:6). ^1^H-NMR (300 MHz, CDCl_3_): δ (as a mixture with 5-epimer) = 5.75 and 5.65 (dd, 1H, *J* = 10.6, 17.3 Hz, 1-H), 5.11–4.65 (m, 4H, 2-H, 3-H), 2.60–2.25 (m, 4H, 6-H, 8-H), 1.78–1.63 (m, 3H, 5-H, 11-H), 1.56–1.48 and 1.36–1.23 (m, 2H, 9-H), 0.91 and 0.90 (s, 3H, 12-H). ^13^C-NMR (150 MHz, CDCl_3_): δ = 13.9, 22.4/26.0, 27.7/29.7, 32.3/32.4, 38.1, 42.7, 56.1/57.4, 112.6/112.7, 113.7/114.3, 143.8/144.7, 144.8/145.7, 211.8/211.9. ESI-MS (*m/z*): 201.1 (M+Na)^+^. HRMS (ESI): calcd for C_12_H_19_O (M+H)^+^, 179.1436, found 179.1400.

## 4. Conclusions

In conclusion, an alternative synthetic route of both diastereomers of (±)-geijerone (**1**) via a 13-step process was achieved starting from the commercially available geraniol (**2**). The Ireland-Claisen rearrangement (**10**→**11**) bearing the *syn*- and *anti*-1,2-dialkenyl carboxylic acid, and the intramolecular Barbier reaction affording the new intramolecular C-C bond (**15**) were the key steps. The newly formed *syn*- and *anti*-1,2-dialkenylcyclohexane strategy used for the synthesis of (±)-geijerone (**1**) and a diastereoisomeric mixture with its 5-epimer allows rapid access to various epimers and analogues of elemene-type products.
